# Enhancing the Utility of Health Related Quality of Life (HRQoL) Assessment Tools in Abdominal Wall Hernia (AWH) Surgery Through Artificial Intelligence (AI): A Framework Proposal

**DOI:** 10.3389/jaws.2025.14952

**Published:** 2025-08-13

**Authors:** Asim Abbas, Maria Luisa Davila Garcia, Srinivas Chintapatla

**Affiliations:** ^1^ York Abdominal Wall Unit, Department of General Surgery, York and Scarborough Teaching Hospitals NHSFT, York Teaching Hospital NHS Foundation Trust, York, United Kingdom; ^2^ School of Computing and Digital Technologies, Sheffield Hallam University, Sheffield, United Kingdom

**Keywords:** quality of life, health related quality of life, PROMs, artificial intelligence, QoL, natural language processing

## Introduction

Abdominal wall hernia (AWH) represents an increasingly prevalent and clinically significant condition, particularly in high-income health systems where surgical volumes for hernia repair continue to rise [1–3]. The evaluation of surgical success has gradually expanded beyond recurrence rates and operative morbidity to include outcomes that are more reflective of patient priorities, such as restoration of function and overall quality of life (QoL) [4, 5]. AWH exerts a broad and often underappreciated impact on patients’ lives. Qualitative research has identified interconnected psychosocial, physical, and socioeconomic domains that are adversely affected [6], including body image [7], mental health [8], sexual and social relationships [9], and employment.

In recognition of these complexities, patient-reported outcome measures (PROMs) have gained prominence as essential tools for capturing the subjective burden of AWH and evaluation of surgical intervention, particularly in Complex Abdominal Wall Reconstruction (CAWR) [10, 11]. Among these, health-related quality of life (HRQoL) assessment tools represent a subset, designed to assess broader impacts of health on physical, psychological, and social functioning. Several instruments have been developed or adapted to quantify domains such as pain, physical limitation, and wellbeing [4]. However, despite increasing uptake in research settings, HRQoL instruments are inconsistently employed in clinical practice, and their interpretive value remains limited [12, 13]. The predominant approach involves scoring and aggregating responses into numerical indices, overlooking nuanced patient experiences, especially in those with complex biopsychosocial needs [14].

Concurrently, artificial intelligence (AI) has demonstrated being a powerful tool in healthcare analytics, offering new capabilities in pattern recognition, data analysis, and predictive analytics. AI refers to digital solutions that simulate cognitive tasks typically requiring human reasoning, including classification, prediction, and pattern recognition. Within this field, machine learning (ML) trains mathematical models to learn from data or past experience in order to make decisions or predictions, while natural language processing (NLP) enables computer algorithms to interpret and analyse human language [15]. In surgical disciplines, AI offers an opportunity to enhance diagnostic imaging, intraoperative decision support, and postoperative risk stratification [16]. More recently, attention has turned to AI’s role in the analysis of PROMs and HRQoL tools, particularly through NLP and ML [17]. In fields such as oncology and orthopaedics, AI is being explored as a potential avenue to enable deeper exploration of patient-reported data, including through the use of free-text narratives and longitudinal symptom trajectories, offering insights that traditional statistical approaches often fail to capture [18, 19]. This presents a timely opportunity for hernia surgery.

### Perspective and Objectives

This paper is positioned as an “Opinion” article, offering a conceptual perspective on how AI could enhance the utility of HRQoL assessment tools in AWH surgery. While existing tools are valuable, their reliance on closed-ended, structured response formats limits their ability to reflect the complexity of patients’ lived experiences, particularly in domains such as body image, mental health, and interpersonal relationships.

This paper has three main aims: to argue current data collection methods in AWH HRQoL tools insufficiently capture the biopsychosocial dimensions of patient experience; to advocate for a shift toward AI-augmented interpretation, with a particular focus on NLP; and to propose a vision for future research and clinical integration, where HRQoL tools serve as dynamic instruments for personalised care.

We support this argument by appraising commonly used AWH HRQoL tools and drawing on other specialties where AI has enriched the analysis of similar data. We conclude with a proposed framework for implementing these approaches.

### Appraising Data Collection Structures in AWH HRQoL Assessment Tools

To support the argument that current HRQoL tools in AWH surgery inadequately capture the complexity of patient experience, we appraise data collection methods and response formats employed by commonly used instruments. In a separate literature review [20], we identified six condition-specific HRQoL assessment tools in AWH populations: Carolinas Comfort Scale (CCS) [21], Hernia-Related Quality of Life Survey (HerQLes) [22], European Registry for Abdominal Wall Hernias QoL instrument (EuraHS-QoL) [23], Activities Assessment Scale (AAS) [24], Abdominal Hernia-Q (AHQ) [25], and Hernia-Related Quality of Life instrument (HERQL) [26].

Our appraisal focuses on response structures (such as Likert scales and binary items) and whether open-ended responses are permitted. Rather than assessing psychometric validity, we examine how design shapes the type and quality of data collected, and the extent to which it permits expression of meaningful patient perspectives.

### Limitations of Current HRQoL and PROM Data in AWH Surgery

The structured appraisal presented in Table 1 highlights a unifying feature across HRQoL assessment tools in AWH surgery: a reliance on fixed-response, closed-ended formats that generate exclusively quantitative data. While practical, they are constrained in their capacity to capture the complexity of individual patient experience [27].

**TABLE 1:** Summary of response structures and data collection methods in AWH-Specific HRQoL assessment tools.

Instrument	Year developed	Country of origin	Target population	Number of items	Response type	Domains covered	Format/administration mode	Summary interpretative notes	Open—ended questions
Activities Assessment Scale (AAS) [23]	2005	United States	Patients with inguinal hernia, particularly for postoperative function	13	6-point ordinal scale (1–5 + 8)	Physical function, mobility, basic and instrumental activities of daily living, and sexual activity	Paper-based questionnaire; Patient self-administered	First tool developed. Designed to quantify physical activity limitation in inguinal hernia patients. Strong for functional profiling	No
Carolinas Comfort Scale (CCS) [20]	2008	United States	Patients with mesh-based hernia repair (inguinal or ventral)	24 (8 activities × 3 sub-questions each)	6-point ordinal scale (0–5)	Mesh sensation, pain, movement limitation during daily activities	Paper-based, self-administered (clinic or post-discharge)	Detects mesh-related complications and activity limitations. Strong clinical utility, reproduced across centres	No
Hernia-Related Quality of Life instrument (HERQL) [25]	2010	Taiwan	Inguinal hernia patients (pre- and post-op)	20	11-point numeric rating scales (0–10), 5-point Likert scales, binary checklists	Pain (rest/activity), activity restriction, GI/urinary/sexual discomfort, QoL impact, economic burden, recurrence concern, satisfaction with surgery	Paper-based questionnaire; Patient self-administered	Multi-domain tool tailored to inguinal hernia with pre/post-operative components. Addresses economic aspects	No
Hernia-Related Quality of Life Survey (HerQLes) [22]	2011	United States	Patients with ventral/incisional hernias	12	6-point Likert scale	Pain, physical function (daily activities), sexual activity, emotional well-being, social isolation, work capacity	Paper-based questionnaire; Patient self-administered	Structured entirely through Likert items. Sexual activity and emotional wellbeing represented	No
European Registry for Abdominal Wall Hernias QoL (EuraHS-QoL) [22]	2012	Belgium/European Initiative	Patients with inguinal and ventral hernias (pre- and post-op)	9 (3 domains × 3 sub-items each)	11-point Numeric Rating Scales (0–10)	Pain, physical restriction, cosmetic concern	Digital and paper-based (registry-compatible); Patient self-administered	Suitable for longitudinal tracking. Concise, registry-integrated tool focused on physical and cosmetic outcomes	No
Abdominal Hernia-Q (AHQ) [24]	2020	USA	Pre and post-operative ventral hernia patients	24	4-point Likert scales (frequency, satisfaction, agreement)	Pain, daily function, sleep, anxiety, body image, satisfaction with care, recovery expectations, patient-clinician communication	Paper-based questionnaire; Patient self-administered	Designed to assess pre- and post-operative hernia experience with patient-centred design. All data is structured and fixed-choice	No

Quantitative HRQoL assessment tools assume that complex biopsychosocial phenomena can be reduced to single numerical scores [28]. Pain is commonly measured on a 0–10 scale, with little or no ability to capture its temporal variability, emotional salience, or relationship to social withdrawal. Similarly, a patient who reports “moderate” difficulty with movement may do so because of mechanical restriction, anxiety about recurrence, or fear of judgment in public. These are qualitatively distinct and clinically relevant.

The absence of open-ended input prevents patients from contextualising their responses [29]. This is problematic for AWH where QoL is multidimensional, with domains such as mental health, body image, and employment, where subjective interpretation and narrative context often matter more than intensity or frequency alone. Without richer explanation, surgeons and researchers make inferences without the patient’s own framing.

This distinction between numerical and narrative formats has also been observed in cancer care. Boomstra et al. (2024) found numerical PROM feedback helped patients take action, while narratives offered emotional support and a sense of recognition [14]. Participants found numbers “cold hard facts,” and stories “easier to grasp,” helping them relate their experiences to others. These findings reinforce the value of narrative data, not as ancillary, but as offering a distinct, complementary perspective.

Composite scores also obscure inter-domain tensions [28]. For example, a patient with high body image distress but low pain may appear to have “moderate” QoL overall, leading to underrecognition of psychosocial burden [30], particularly in populations at risk of underreporting due to stigma or stoicism.

Structured HRQoL assessment tools offer limited insight into longitudinal change. Applied cross-sectionally, they fail to capture evolving trajectories. Without temporally sensitive narratives, surgeons lack cues that distinguish recovery from stagnation or decline; information that could otherwise guide escalation of care [31].

These limitations are not merely technical but also philosophical [28, 29]. The reduction of subjective experiences to numerical values assumes that complex human experiences can be entirely quantified, overlooking the nuanced and evolving nature of individual experiences, particularly in illness. Philosophers, like Havi Carel, emphasise the importance of acknowledging the lived experience of illness [32], arguing that purely objective measures can fail to capture the patient’s perspective. In the context of HRQoL assessment tools, standardised metrics may lead to the loss of rich patient narratives [14]. This critique aligns with calls for a pragmatic epistemology in HRQoL tool development [28].

### Applications in Other Specialties

Other medical specialties have begun addressing similar challenges by integrating AI techniques, particularly NLP and ML, into analysis of patient-reported data.

In clinical settings, NLP allows free-text responses - such as open-ended survey answers, interviews, or electronic health record (EHR) notes - to be analysed for themes, sentiment, and clinically relevant content. Unlike numerical scores, narratives capture temporality, tone, and context [33–35], all essential for understanding complex psychosocial domains such as pain, fatigue, mental distress, or body image.

A 2024 systematic review by Sim et al. synthesized 22 oncology studies using NLP to extract and analyse unstructured PROMs [36]. These studies analysed narrative data from progress notes, discharge summaries, and consultations. Most employed a multi-stage process: preprocessing (such as tokenisation, lemmatisation, and removal of stop words), feature extraction using term frequency–inverse document frequency (TF-IDF) or named entity recognition, and predictive modelling using ML architectures. Some also revealed latent constructs, such as pain interference and fatigue in childhood cancer survivors, not captured by structured tools. Notably, this approach reused existing clinical text, reducing additional surveys and minimising administrative burden.

Coleman et al. (2025) developed an NLP pipeline to detect PROM documentation in over 377,000 unstructured EHR notes in Veterans Health Administration (VHA) chiropractic clinics [37]. They compared rule-based models (using medspaCy library) with ML models such as bag-of-words and neural networks. Their primary objective was to categorise clinic notes by whether they documented PROM usage. The rule-based system outperformed ML classifiers (90.3% precision, 99.5% recall, F1 score 94.7%). Only 17% of notes documented PROM use, highlighting NLP’s promise in retrospective audit for challenges such as inconsistent documentation.

Another example is the Artificial Intelligence Patient-Reported Experience Measure (AI-PREM) developed by van Buchem et al. (2022) in the Netherlands [38]. AI-PREM consists of five open-ended questions, worded to be accessible to patients and structurally suited to NLP-based analysis. Responses were analysed via a two-stage NLP pipeline: sentiment classification using a fine-tuned BERT model (F1 = 0.97), and topic modelling via non-negative matrix factorisation (NMF). The system achieved 90% concordance with expert manual coding and presented outputs in a dashboard that linked topic summaries to individual comments, preserving narrative richness while supporting clinical interpretation.

## Discussion

AWH surgery is uniquely positioned to benefit from NLP-enhanced HRQoL assessment tools. The biopsychosocial burden of AWH spans across domains poorly captured by traditional instruments, including body image concerns, return-to-work challenges, and psychological distress. Qualitative research shows these themes often emerge through narrative expression rather than scale-based responses [6]. Furthermore, decisions to pursue complex abdominal wall reconstruction are highly individualised and preference-sensitive. Surgeons must balance technical feasibility against subjective expectations and priorities. NLP-enhanced HRQoL tools could offer a richer, more interpretable view of how patients frame their concerns and aspirations, informing shared decision-making and preoperative counselling.

Rather than replacing traditional instruments, we encourage a hybrid approach, combining structured data with narrative input, analysed using AI, to capture the full depth of patient experience. This requires both a conceptual shift and a practical implementation framework. At the design stage, HRQoL tools should include open-ended questions, particularly in domains where individual variation is high and structured items may fail to reflect whole patient reality, such as body image, emotional distress, or interpersonal strain. These questions must be constructed with specificity to enable meaningful NLP processing [39]. This “narrative-enabled design” should be paired with co-development by patients, surgeons, and researchers to ensure prompts are clinically relevant and emotionally safe.

For NLP-compatibility, instruments must support semantic disambiguation and topic extraction [40]. Outputs must be intelligible to users, with results displayed in layered, interpretable formats that allow clinicians to move between population-level insights, such as overall sentiment trends or dominant themes, and individual narratives. Ideally, these outputs are integrated into a dashboard or platform that supports real-time engagement with data [40, 41].

Transparency and validation are vital. NLP models must be trained on high-quality, annotated datasets with iterative evaluation. Human-coded benchmarks are essential to assess concordance with machine-derived themes and sentiment. Ethical implementation requires patient consent, handling patient narratives with respect, data privacy protections, and safeguards against algorithmic bias - these must be embedded in the design from the outset [42].

To meaningfully integrate AI into HRQoL assessment in AWH surgery, future tools must be guided by a clear set of design principles and a structured framework for implementation. At the core of this shift is the recognition that open-ended, narrative responses, when appropriately captured and analysed, can enrich understanding in ways that complement traditional quantitative instruments.

We propose a four-stage conceptual framework: The first stage is data capture, in which HRQoL assessment tools incorporating open-text fields are delivered through digital platforms at key points in the clinical pathway - preoperatively, postoperatively, and longitudinally. The second stage is the NLP pipeline, where responses are processed using validated models for sentiment analysis, topic modelling, and symptom or entity recognition. The third stage is interpretation, in which outputs are synthesised into formats digestible to surgeons, researchers, and patients. The final stage is integration, where findings inform personalised care plans, multidisciplinary team discussions, research, and/or registry-linked initiatives.

This shift reflects a methodological and philosophical evolution, toward a pragmatic epistemology that values both standardised metrics and patients’ narrative accounts as complementary resources, ripe for hermeneutic excavation.

## References

[1] Sureshkumar S Sudharsanan S Vijayakumar C Anandhi A. Abdominal Wall Reconstruction: Advances in the Last Decade. Int J Adv Med Health Res (2024) 11(1):4–14. 10.4103/ijamr.ijamr_310_23

[2] Poulose BK Shelton J Phillips S Moore D Nealon W Penson D Epidemiology and Cost of Ventral Hernia Repair: Making the Case for Hernia Research. Hernia J Hernias Abdom Wall Surg (2012) 16(2):179–83. 10.1007/s10029-011-0879-9 21904861

[3] Ma Q Jing W Liu X Liu J Liu M Chen J. The Global, Regional, and National Burden and Its Trends of Inguinal, Femoral, and Abdominal Hernia from 1990 to 2019: Findings from the 2019 Global Burden of Disease Study - a cross-sectional Study. Int J Surg Lond Engl (2023) 109(3):333–42. 10.1097/JS9.0000000000000217 PMC10389329 37093073

[4] Hill S Bullock J Sanders DL. Quality of Life with a Hernia-A Novel Patient Led Study. J Abdom Wall Surg JAWS (2023) 2:11214. 10.3389/jaws.2023.11214 38312408 PMC10831678

[5] van Ramshorst GH Eker HH Hop WCJ Jeekel J Lange JF. Impact of Incisional Hernia on health-related Quality of Life and Body Image: A Prospective Cohort Study. Am J Surg (2012) 204(2):144–50. 10.1016/j.amjsurg.2012.01.012 22579232

[6] Smith OA Mierzwinski MF Chitsabesan P Chintapatla S. Health-Related Quality of Life in Abdominal Wall Hernia: Let’s Ask Patients what Matters to Them? Hernia (2022) 26(3):795–808. 10.1007/s10029-022-02599-6 35412193 PMC9003180

[7] Smith O Mierzwinski M Oliver-Jenkins V MacLeod T Chitsabesan P Chintapatla S. Novel Insights into Patient’s Thoughts About Their Body Image in Abdominal Wall Hernia. Hernia (2023) 28(1):43–51. 10.1007/s10029-023-02896-8 37910297

[8] Smith Oa. M Mierzwinski M McVey J Chitsabesan P Chintapatla S. Abdominal Wall Hernia and Mental Health: Patients Lived Experiences and Implications for Patient Care. Hernia J Hernias Abdom Wall Surg (2023) 27(1):55–62. 10.1007/s10029-022-02699-3 PMC9595579 36284067

[9] Smith O Abbas A Mierzwinski M Oliver-Jenkins V Chitsabesan P Chintapatla S (2025). The Impact of Abdominal Wall Hernia (AWH) on Patients’ Social and Sexual Relationships: A Qualitative Analysis. Hernia 29, 234. 10.1007/s10029-025-03414-8 PMC12267305 40668307

[10] van Veenendaal N Poelman MM van den Heuvel B Dwars BJ Schreurs WH Stoot JHMB Patient-Reported Outcomes After Incisional Hernia Repair. Hernia J Hernias Abdom Wall Surg (2021) 25(6):1677–84. 10.1007/s10029-021-02477-7 PMC8613099 34338938

[11] Langbach O Bukholm I Jš B Røkke O. Long-Term Quality of Life and Functionality After Ventral Hernia Mesh Repair. Surg Endosc (2016) 30(11):5023–33. 10.1007/s00464-016-4850-9 26969665

[12] Gram-Hanssen A Christophersen C Rosenberg J. Results from patient-reported Outcome Measures Are Inconsistently Reported in Inguinal Hernia Trials: A Systematic Review. Hernia (2022) 26(3):687–99. 10.1007/s10029-021-02492-8 34480660

[13] Grove TN Muirhead LJ Parker SG Brogden DRL Mills SC Kontovounisios C Measuring Quality of Life in Patients with Abdominal Wall Hernias: A Systematic Review of Available Tools. Hernia J Hernias Abdom Wall Surg (2021) 25(2):491–500. 10.1007/s10029-020-02210-w PMC8055629 32415651

[14] Boomstra E Hommes S Vromans RD Van Der Burg S Schrijver AM Wouters MWJM “Numbers Call for Action, Personalized Narratives Provide Support and Recognition”: A Qualitative Assessment of Cancer Patients’ Perspectives on patient-reported Outcome Measures (Proms) Feedback with Narratives. J Cancer Surviv (2024). 10.1007/s11764-024-01663-7 PMC12988888 39320669

[15] Bajwa J Munir U Nori A Williams B. Artificial Intelligence in Healthcare: Transforming the Practice of Medicine. Future Healthc J (2021) 8(2):e188–94. 10.7861/fhj.2021-0095 34286183 PMC8285156

[16] Varghese C Harrison EM O’Grady G Topol EJ. Artificial Intelligence in Surgery. Nat Med (2024) 30(5):1257–68. 10.1038/s41591-024-02970-3 38740998

[17] Wójcik Z Dimitrova V Warrington L Velikova G Absolom K. Using Artificial Intelligence to Predict Patient Outcomes from patient-reported Outcome Measures: A Scoping Review. Health Qual Life Outcomes (2025) 23(1):37. 10.1186/s12955-025-02365-z 40217230 PMC11987430

[18] Krepper D Cesari M Hubel NJ Zelger P Sztankay MJ. Machine Learning Models Including patient-reported Outcome Data in Oncology: A Systematic Literature Review and Analysis of Their Reporting Quality. J Patient-rep Outcomes (2024) 8(1):126. 10.1186/s41687-024-00808-7 39499409 PMC11538124

[19] Kunze KN Madjarova S Jaykumar P Nwachukwu BU. Challenges and Opportunities for the Use of Patient-Reported Outcome Measures in Orthopaedic Pediatric and Sports Medicine Surgery. J Am Acad Orthop Surg (2023) 31:e898–e905. 10.5435/JAAOS-D-23-00087 37279168

[20] Oxley C Smith O Abbas A A critical COSMIN-informed scoping review of complex abdominal wall hernia quality of life tools: making a case for patient-driven tool development. Hernia (2025) 29, 219. 10.1007/s10029-025-03399-4 40601019 PMC12222306

[21] Heniford TB Walters AL Lincourt AE Novitsky YW Hope WW Kercher KW. Comparison of Generic Versus Specific quality-of-life Scales for Mesh Hernia Repairs. J Am Coll Surg (2008) 206(4):638–44. 10.1016/j.jamcollsurg.2007.11.025 18387468

[22] Krpata DM Schmotzer BJ Flocke S Jin J Blatnik JA Ermlich B Design and Initial Implementation of Herqles: A Hernia-Related quality-Of-life Survey to Assess Abdominal Wall Function. J Am Coll Surg (2012) 215(5):635–42. 10.1016/j.jamcollsurg.2012.06.412 22867715

[23] Muysoms F Campanelli G Champault GG DeBeaux AC Dietz UA Jeekel J Eurahs: The Development of an International Online Platform for Registration and Outcome Measurement of Ventral Abdominal Wall Hernia Repair. Hernia J Hernias Abdom Wall Surg (2012) 16(3):239–50. 10.1007/s10029-012-0912-7 PMC3360853 22527930

[24] McCarthy M Jonasson O Chang CH Pickard SA Giobbie-Hurder A Gibbs J Assessment of Patient Functional Status After Surgery. J Am Coll Surg (2005) 201(2):171–8. 10.1016/j.jamcollsurg.2005.03.035 16038812

[25] Mauch JT Enriquez FA Shea JA Barg FK Rhemtulla IA Broach RB The Abdominal Hernia-Q: Development, Psychometric Evaluation, and Prospective Testing. Ann Surg (2020) 271(5):949–57. 10.1097/SLA.0000000000003144 30601257

[26] Huang CC Tai FC Chou TH Lien HH Jeng JY Ho TF Quality of Life of Inguinal Hernia Patients in Taiwan: The Application of the Hernia-specific Quality of Life Assessment Instrument. PLoS One (2017) 12(8):e0183138. 10.1371/journal.pone.0183138 28817703 PMC5560705

[27] Greenhalgh J Flynn R Long AF Tyson S. Tacit and Encoded Knowledge in the Use of Standardised Outcome Measures in Multidisciplinary Team Decision Making: A Case Study of in-patient Neurorehabilitation. Soc Sci Med (2008) 67(1):183–94. 10.1016/j.socscimed.2008.03.006 18403078

[28] Meadows KA. A Philosophical Perspective on the Development and Application of patient-reported Outcomes Measures (Proms). Qual Life Res (2022) 31(6):1703–9. 10.1007/s11136-021-03016-8 34657279

[29] Neale J Strang J. Philosophical Ruminations on Measurement: Methodological Orientations of Patient Reported Outcome Measures (PROMS). J Ment Health (2015) 24(3):123–5. 10.3109/09638237.2015.1036978 25989489

[30] Newson JJ Sukhoi O Thiagarajan TC. MHQ: Constructing an Aggregate Metric of Population Mental Wellbeing. Popul Health Metr (2024) 22(1):16. 10.1186/s12963-024-00336-y 39020379 PMC11256620

[31] Snyder CF Aaronson NK Choucair AK Elliott TE Greenhalgh J Halyard MY Implementing patient-reported Outcomes Assessment in Clinical Practice: A Review of the Options and Considerations. Qual Life Res (2012) 21(8):1305–14. 10.1007/s11136-011-0054-x 22048932

[32] Carel H. Phenomenology as a Resource for Patients. J Med Philos (2012) 37(2):96–113. 10.1093/jmp/jhs008 22474139

[33] Palla I Turchetti G Polvani S. Narrative Medicine: Theory, Clinical Practice and Education - a Scoping Review. BMC Health Serv Res (2024) 24(1):1116. 10.1186/s12913-024-11530-x 39334149 PMC11428871

[34] Semyonov-Tal K Lewin-Epstein N. The Importance of Combining open-ended and closed-ended Questions when Conducting Patient Satisfaction Surveys in Hospitals. Health Policy OPEN (2021) 2:100033. 10.1016/j.hpopen.2021.100033 37383512 PMC10297822

[35] Hansen K Świderska A. Integrating open- and closed-ended Questions on Attitudes Towards Outgroups with Different Methods of Text Analysis. Behav Res Methods (2024) 56(5):4802–22. 10.3758/s13428-023-02218-x 37845422 PMC11289311

[36] Sim JA Huang X Horan MR Baker JN Huang IC. Using Natural Language Processing to Analyze Unstructured patient-reported Outcomes Data Derived from Electronic Health Records for Cancer Populations: A Systematic Review. Expert Rev Pharmacoecon Outcomes Res (2024) 24(4):467–75. 10.1080/14737167.2024.2322664 38383308 PMC11001514

[37] Coleman BC Corcoran KL Brandt CA Goulet JL Luther SL Lisi AJ. Identifying Patient-Reported Outcome Measure Documentation in Veterans Health Administration Chiropractic Clinic Notes: Natural Language Processing Analysis. JMIR Med Inform (2025) 13:e66466. 10.2196/66466 40173367 PMC12038758

[38] Van Buchem MM Neve OM Kant IMJ Steyerberg EW Boosman H Hensen EF. Analyzing Patient Experiences Using Natural Language Processing: Development and Validation of the Artificial Intelligence Patient Reported Experience Measure (AI-PREM). BMC Med Inform Decis Mak (2022) 22(1):183. 10.1186/s12911-022-01923-5 35840972 PMC9284859

[39] Tamang S Humbert-Droz M Gianfrancesco M Izadi Z Schmajuk G Yazdany J. Practical Considerations for Developing Clinical Natural Language Processing Systems for Population Health Management and Measurement. JMIR Med Inform (2023) 11:e37805. 10.2196/37805 36595345 PMC9846439

[40] Aramaki E Wakamiya S Yada S Nakamura Y. Natural Language Processing: From Bedside to Everywhere. Yearb Med Inform (2022) 31(1):243–53. 10.1055/s-0042-1742510 35654422 PMC9719781

[41] Chapman WW Nadkarni PM Hirschman L D’Avolio LW Savova GK Uzuner O. Overcoming Barriers to NLP for Clinical Text: The Role of Shared Tasks and the Need for Additional Creative Solutions. J Am Med Inform Assoc JAMIA (2011) 18(5):540–3. 10.1136/amiajnl-2011-000465 21846785 PMC3168329

[42] Petit-Jean T Gérardin C Berthelot E Chatellier G Frank M Tannier X Collaborative and privacy-enhancing Workflows on a Clinical Data Warehouse: An Example Developing Natural Language Processing Pipelines to Detect Medical Conditions. J Am Med Inform Assoc JAMIA (2024) 31(6):1280–90. 10.1093/jamia/ocae069 38573195 PMC11105139

